# The association between *BMP4* gene polymorphism and its serum level with the incidence of LVH in hypertensive patients

**DOI:** 10.1186/s12967-014-0368-x

**Published:** 2015-01-16

**Authors:** GL Gu, QY Yang, RL Zeng, XL Xu

**Affiliations:** Department of cardiovascular diseases, Jiangyin Hospital of traditional Chinese medicine affiliated Nanjing University of Chinese Medicine, Jiangyin, 214400 Jiangsu China; Department of cardiovascular diseases, Wuxi Hospital of traditional Chinese medicine, Jiangyin, 214400 Jiangsu China; Department of cardiovascular diseases, The People’s Hospital of Jiangyin, Jiangyin, 214400 Jiangsu China; Department of Cardiothoracic Surgery, Huashan Hospital, Fudan University, Shanghai, 214400 PR China

**Keywords:** Bone morphogenic proteins 4, Essential hypertension, Left ventricular hypertrophy, Small interfering RNA

## Abstract

**Background:**

Bone morphogenic proteins 4 (BMP4) is associated with cardiac remodeling under different conditions. However, the role of BMP4 and its gene polymorphism in the incidence of left ventricular hypertrophy (LVH) in hypertensive patients remains unknown.

**Methods:**

A total of 1265 patients diagnosed with essential hypertension (EH) were recruited. Patients were assigned to LVH+ (n = 420) and LVH- (n = 845) groups. serum BMP4 level was measured and two single nucleotide polymorphism (SNPs) polymorphisms, *6007C > T* and *-5826G > A* of *BMP4* gene were genotyped. We also inhibited the BMP4 by small interfering RNA (siRNA). The effect of BMP4 on the hypertrophic response in Human Cardiomyocytes AC16 cells was studied.

**Results:**

We found that the 6007C > T polymorphism of the *BMP4* gene and the serum BMP4 level were significantly associated with the risk to develop LVH. With TT as reference, multivariate logistic regression analysis showed the 6007CC genotype carriers had a higher susceptibility to LVH incidence (adjusted OR = 2.65, 95% CI: 1.63-4.31, adjusted P < 0.001). Our *in vitro* study shows that the BMP4 inhibition in cardiomyocyte by si-RNA technique significantly decreased the Ang II induced cardiomyocyte size and protein content per cell, indicating the importance of BMP4 in the cardiomyocyte hypertrophy.

**Conclusion:**

Collectively, our data suggest that both the 6007C > T of the *BMP4* gene and the serum BMP4 level may be used as potential marker for LVH incidence among the EH patients.

**Electronic supplementary material:**

The online version of this article (doi:10.1186/s12967-014-0368-x) contains supplementary material, which is available to authorized users.

## Background

Left ventricular hypertrophy (LVH) is one of the major complication of high blood pressure, which is regarded as an independent risk factor for cardiovascular morbidity and mortality [1-4]. The established risk factors responsible for LVH includes blood pressure level, duration of hypertension, age, obesity, diet, and pharmacologic treatment [5,6]. In addition, a large body of evidence shows that the individual genetic background predisposes the LVH incidence [7-9]. Identifying the key factor mediating pathological cardiac hypertrophy is critically important for developing the strategy to protect against heart failure.

BMP4 is a member of bone morphogenic proteins (BMP) superfamily, regulating osteoblast differentiation and bone formation [10-13]. In addition, BMP4 is a mechanosensitive and proinflammatory gene, and it induces endothelial cell apoptosis, and endothelium dysfunction [14,15]. BMP4 induces cardiomyocyte differentiation and promotes cardiomyocyte apoptosis after ischemia-reperfusion injury–induced myocardial infarction [16,17]. The association between BMP4 and cardiac remodeling is recently reported. BMP4 is expressed in human and mouse hearts and recombinant BMP4 protects adult mouse cardiomyocytes against hypoxia-reoxygenation injury [18]. BMP4 induces cardiomyocyte hypertrophy and apoptosis through increasing nadph oxidase 4 expression and reactive oxygen species-dependent pathways [19].

Genetic variants in *BMP4*, in the form of single nucleotide polymorphisms (SNPs), may result in a qualitative or quantitative change in the local production of *BMP4* or in its effectiveness via its cognate receptor [20]. Although many mutations within *BMP4* leading to various phenotypes have been reported [21], the single nucleotide polymorphism of 6007C > T (rs17563) of *BMP4* is the only identified polymorphism in the coding region [22]. To date, there is no study regarding the association between BMP4 gene polymorphism and LVH incidence.

Given the association between BMP4 and cardiac remodeling [19], we postulate that the BMP4 gene polymorphism may also affect the cardiac remodeling. In this study, we enrolled essential hypertentive patients with and without LVH, to test this hypothesis.

## Methods

### Enrollment

A total of 1265 patients diagnosed with EH were recruited in our hospital from July 2007 to March 2012. All patients were assigned into LVH+ (EH with LVH) and LVH- (EH without LVH) groups based on the presence or absence of LVH. A complete medical history was obtained from all subjects, including diabetes mellitus (DM), alcohol intake, cigarette smoking, weight, height, body mass index (BMI), systolic blood pressure (SBP), and diastolic blood pressure (DBP). The study protocol was approved by the ethics committee of Nanjing University of Chinese Medicine. All patients provided an informed written consent. We measured total cholesterol (TC), total triglyceride (TG), high-density lipoprotein cholesterol (HDL-C), low-density lipoprotein cholesterol (LDL-C), creatine kinase (CK), creatine kinase-MB (CK-MB), troponin T (TNT), fasting blood glucose (FBG), blood urea nitrogen (BUN), and serum creatinine [23].

### Measurement of LVH

The echocardiography was performed in three cardiac cycles at end diastole and end systole, by the two investigators who were blind to the genotypes of the patients using a Hewlett–Packard imaging system (Sonos 2500 model, California). All measurements LV mass (LVM) was calculated at end-diastole using the formula: 0.8 × 1.04[(IVSd + LVIDD + PWTd) − LVIDD] + 0.6 (IVSd: interventricular septal thickness, PWTd: posterior wall thickness, LVIDD: LV end-diastolic internal dimension), which yields values closely related (R = 0.90) to necropsy LV weight. LVM was divided by height 2.7 to obtain LVMI (LVM index). LVH was defined as LVMI > 49.2 g/2.7 m for men and >46.7 g/2.7 m for women [13].

### Genotyping of *BMP4* gene

DNA was extracted from peripheral whole blood using a Qiagen DNA Isolation Kit (Qiagen, Valencia, CA, USA). The specific product of the *BMP4* gene was amplified by polymerase chain reaction (PCR) with primers 5’-biotion-TGAAGGCAAGATGTCTGA- CACA-3’ (forward) and 5’-CCTTCCTGCATTTCTATCCTA-3’ (reverse) for -5826G > A (rs1957860), and 5’-ATTGCCCAACCCTGAGCTATC-3’ (forward) and 5’-biotin-TGGGGGCTTCATAACCTC-3’ (reverse) for 6007C > T (rs17563). Reactions were performed in a total volume of 20 μl. The thermocycling procedure consisted of initial denaturation at 95°C for 3 minutes, 35 cycles of denaturation at 94°C for 30 seconds, annealing at 60°C for 40 seconds, extension at 72°C for 1 minute and a final extension at 72°C for 10 minutes. The PCR products were analyzed by electrophoresis on 1% agarose gel [24].

### Serum BMP4 and other cytokine Measurement

Serum BMP4 was measured using the BMP4 Duoset enzyme linked immunoassay kit (R & D Systems, Minneapolis, MN) according to manufacturer’s instructions. Samples were assayed in duplicate. The interassay coefficient of variance was < 14% and the detection limit of the assay was 15.6 pg/mL [25]. Serum cystatin-C was measured using an immunoturbidimetric method (Roche Diagnostics, Indianapolis, IN) following the manufacturer’s recommendations. The PIIINP levels were assayed using enzyme linked immunosorbent assay kits (Nivelles, Belgium). The Serum hs-CRP level was measured by using the high sensitivity CRP Vario assay (Abbott Laboratories, Abbott Park, Illinois).

### Rat cardiomyocytes culture

The whole hearts from neonate rats were isolated, minced and rinsed in hood by using by using a device invented by Xuwei Hou (patent number: CN 101955884 A and CN 101955884 B). Briefly, whole hearts from neonate rats (age less than 3 days) were isolated, minced and rinsed in hood. Five to six cycles of digestion using collagenase (95 U/ml, Sigma Aldrich, Bornem, Belgium) and 0.6 U/ml of pancreatin (Sigma Aldrich, Bornem, Belgium) were performed. After cell plating on non-coated culture dishes for 2 h to allow differential attachment of non-myocyte cells, the remaining cell suspension containing neonatal cardiomyocytes was collected, counted, and seeded at 5*104 cells/cm2 in collagen I-coated culture dishes. The medium was replaced after 72 h and cardiomyocytes were allowed to reach confluence before use. The isolated cardiomyocytes were pooled and resuspended in mixed medium containing DMEM and M199 (volume ratio: 4:1) and 10% fetal bovine serum (FBS) in incubator at37 degree with 5% CO2 [26].

### BMP4 siRNA transfection

Cultured cells were expanded to reach a 70% confluence. Then cells were maintained in antibiotic free mixed medium containing DMEM and M199 for 24 hour before si-RNA transfection. Nonspecific control siRNA or BMP4 siRNA (Santa Cruz Biotechnology, USA) was transfected by siLentFect Lipid Reagent (Bio-Rad, Hercules, CA, USA) for 48 hours in an incubator at 37 degree with 5% CO2, according to the manufacturer’s instructions.

### Hypertrophy assays

Cardiomyocytes were stimulated with 10(−6) mol/L angiotensin (Ang) II for 48 h. Then the cells were fixed in 4% paraformaldehyde, stained with FITC-conjugated Phalloidin (Sigma) for 30 min and mounted in Vectashield with 4’,6-diamidino-2-phenylindole (Vector Laboratories, Peterborough, UK). Cellular hypertrophy was evaluated by measuring cardiomyocytes and cardiac fibroblasts surfaces using a digital image analysis system (Leica QwinV3, Leica Microsystems Ltd., Cambridge, UK). Five random fields (with approximately 10 to 15 cells per field) from every sample were averaged and expressed as μm2/cell. All experiments were repeated three times.

### Measurement of Protein Synthesis in cardiomyocytes

The AngII treated cells were trypsinized and counted using a cell counting chamber (Beckman Coulter, Fullerton, CA, USA) and then lysed. A total of 1*10(5) cells were used for the Protein Synthesis measurement. The cell lysates were prepared to determine protein content by Bradford protein assay. Then the protein synthesis of cells was determined by dividing the total amount of protein by the number of cells, namely, protein per cell [27].

### Statistical analysis

Data on quantitative characteristics are expressed as means ± SD. Data on qualitative characteristics are expressed as percent values or absolute numbers, as indicated. Differences in demographic characteristics and vascular risk factors between patients and controls were compared by using Student’s t test for continuous variables and the χ2 test for all categorical variables. Tests for Hardy-Weinberg equilibrium for the gene polymorphisms were conducted using χ2 tests. Genotypes and allele frequencies were compared by χ2 analysis. Multivariate logistic regression analysis was used to determine the influence of *BMP4* polymorphisms on LVH risk, with controlling potential confounding risk variables. A forward stepwise (Likelihood Ratio) procedure was used for multivariable analysis. Data were analyzed with the SPSS 13.0 package (SPSS Inc) and results were considered statistically significant at P < 0.01 using a 2-tailed test.

## Results

The clinical and biochemical characteristics of all subjects are shown in Table 1. A total of 420 patients were assigned to the LVH+ group and 845 to the LVH- group. There were no significant differences in age, sex, alcohol intake, BMI, TG, TC, HDL, LDL and sCr between LVH+ and LVH- groups. In addition, the treatment time of ACEI/ARBs was similar between two groups. The mean LVMI in LVH+ group was 51.3 ± 3.8 g/m2 while that in LVH− group was 43.3 ± 4.1 g/m2 (P < 0.001, Table 1).

**Table 1:** **Clinical and biochemical characteristics of all subjects**

**Variables**	**LVH+ N = 420**	**LVH− N = 845**	**P value**
Age (years)	52.5 ± 6.6	51.8 ± 8.4	NS
Male (n, %)	67.5%	68.2%	NS
Alcohol intake (n, %)	32.8%	30.5%	NS
Smoker (n, %)	46.7%	26.4.1%	0.011
BMI (kg/m2)	24.4 ± 2.2	23.8 ± 3.5	NS
SBP (mmHg)	152.9 ± 8.0	149.6 ± 11.5	NS
DBP (mmHg)	87.8 ± 7.9	89.5 ± 10.6	NS
TG (ng/dl)	5.2 ± 2.8	5.1 ± 2.3	NS
TC (ng/dl)	1.6 ± 0.3	1.6 ± 0.4	NS
HDL-C (ng/dl)	1.3 ± 0.8	1.7 ± 0.5	NS
LDL-C (ng/dl)	2.5 ± 0.8	2.6 ± 0.5	NS
sCr (mmol/l)	101.6 ± 11.5	90.8 ± 12.7	NS
Duration since EH diagnosis (months)	24.4 ± 3.9	23.7 ± 5.2	NS
Therapy protocol			
ACEI (%)	36.7	35.9	NS
Time (month)	17.7 ± 3.2	16.8 ± 4.8	NS
ARB (%)	28.9	28.9	NS
Time (month)	12.8 ± 2.2	12.4 ± 1.8	NS
CCB (%)	33.2	32.6	NS
Time (month)	13.5 ± 3.1	13.4 ± 1.9	NS
Diuretics (%)	6.8	6.7	NS
Time (month)	8.7 ± 2.1	8.8 ± 2.6	NS
β-blocker (%)	23.7	24.1	NS
Time (month)	16.2 ± 3.0	16.0 ± 2.3	NS
Others (%)	22.7	21.8	NS
Time (month)	7.7 ± 1.2	7.8 ± 0.9	NS

Genotype frequencies of *BMP4* polymorphisms in LVH+ (N = 420) and LVH- (N = 845) groups were found to be in Hardy–Weinberg equilibrium (all P > 0.05). The genotype and the allele frequencies of -5826G > A were not significantly different between two groups. However, the genotypes and allele frequency of 6007C > T were significantly different between LVH+ and LVH- patients. The 6007CC genotype was much more prevalent in LVH+ patients than LVH- patients (33.10%% vs. 19.17%, Table 2). With TT as reference, multivariate logistic regression analysis showed the 6007CC genotype carriers had a higher susceptibility to LVH incidence (adjusted OR = 2.33, 95% CI: 1.67-3.25, adjusted P < 0.001) with adjustment for age, sex, smoke status, TG, TC, BP level, serum hs-CRP, serum OPN levels. The 6007C allele conferred a higher risk for LVH development (adjusted OR = 1.54, 95% CI: 1.30-1.82, P < 0.001) compared with T allele carriage.

**Table 2:** **The genotype frequencies for BMP-4 polymorphisms between LVH+ and LVH- groups**

		***LVH+***		***LVH-***		***Adjusted OR***	***95% CI***		***Adjusted P***	
*−5826G > A*		*N = 420*		*N = 845*	*%*					
	6007C > T *TT*	90	21.43%	243	28.76%	1.00				
	*CT*	191	45.48%	436	51.60%	1.18	0.88	1.59	0.265	0.259
	*CC*	139	33.10%	166	19.64%	2.26	1.62	3.15	0.000	0.000
	*T*	371	44.17%	922	54.56%	1.00				
	*C*	469	55.83%	768	45.44%	1.52	1.28	1.79	0.000	0.000
*6007C > T*	−5826G > A *TT*	107	25.48%	201	23.79%	1.00				
	*CT*	205	48.81%	437	51.72%	0.88	0.66	1.17	0.388	0.542
	*CC*	108	25.71%	207	24.50%	0.98	0.70	1.36	0.905	0.524
	*T*	419	49.88%	839	49.64%	1.00				
	*C*	421	50.12%	851	50.36%	0.99	0.84	1.17	0.911	0.517

**Figure 1:**
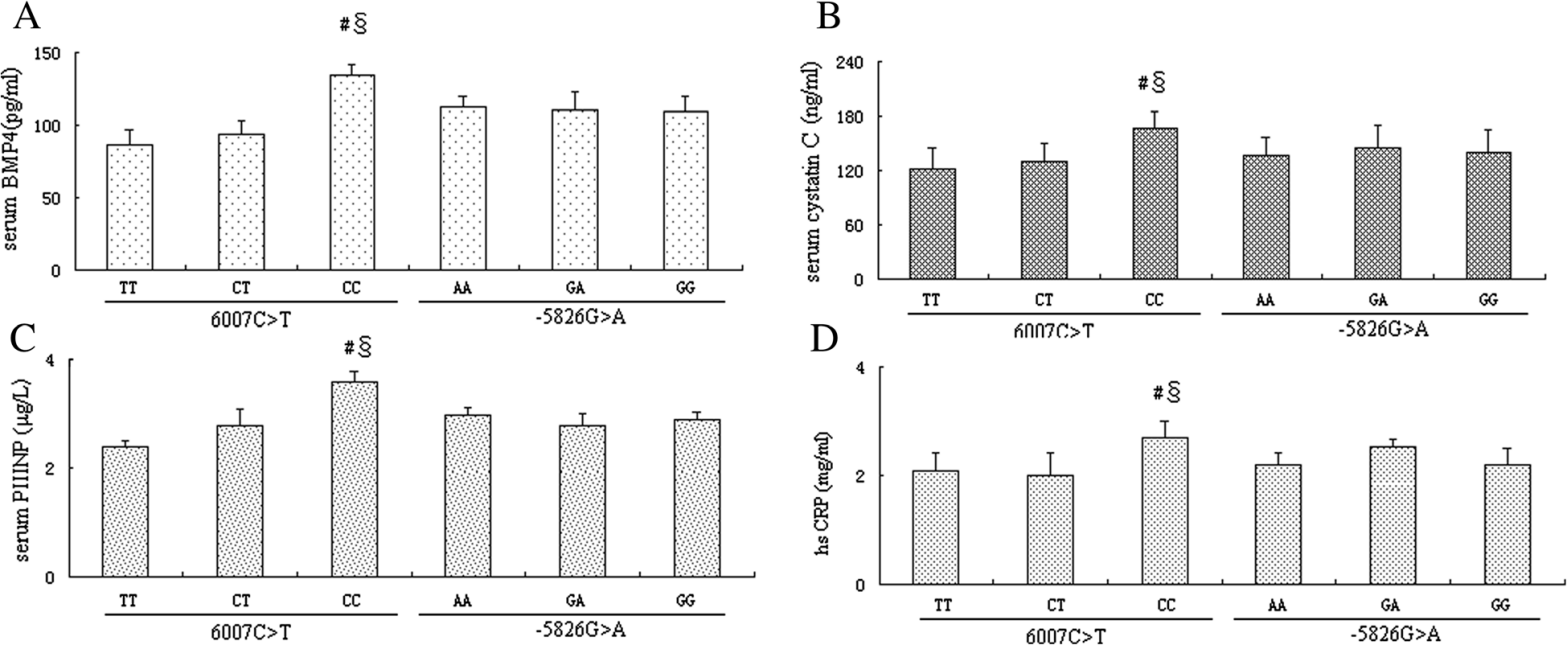
**The association between the BMP4 genotype and the serum BMP4, cystatin C, PIIINP and hs-CRP levels.** The 6007CC genotype carries had higher serum BMP4 levels than CT and TT carriers **(A)**. Moreover, the 6007C > T is also significantly associated with the serum cystatin C **(B)**, PIIINP **(C)** and hs-CRP **(D)** levels (Figure B-D, all P < 0.001).

We next analyzed the association between the *BMP4* genotype and the serum BMP4 levels. We found that the 6007C > T significantly affect the serum BMP4 level (Figure 1A). The 6007CC genotype carries had higher serum *BMP4* levels than CT and TT carriers (P < 0.001). Moreover, the 6007C > T is also significantly associated with the serum cystatin C, PIIINP and hs-CRP levels (Figure 1B-D, all P < 0.001). In contrast, the -5826G > A genotype did not affect the serum BMP4, cystatin C, PIIINP and hs-CRP levels.

We next investigated the correlation between serum BMP4, serum cystatin C and serum hs-CRP levels with the LVMI in patients. We observed a significantly correlation between serum BMP4 and LVMI (r = 0.534, P < 0.001). Likewise, the serum cystatin C and PIIINP levels are also correlated to the LVMI (r = 0.542, P = 0.002; r = 0.688, P < 0.001respectively). No correlation was found between serum hsCRP and LVMI in this study (r = 0.162, P = 0.061). The serum BMP4 level was significantly correlated to the serum cystatin C, and PIIINP (r = 0.421, P = 0.002; r = 0.582, P < 0.001, respectively).

**Figure 2:**
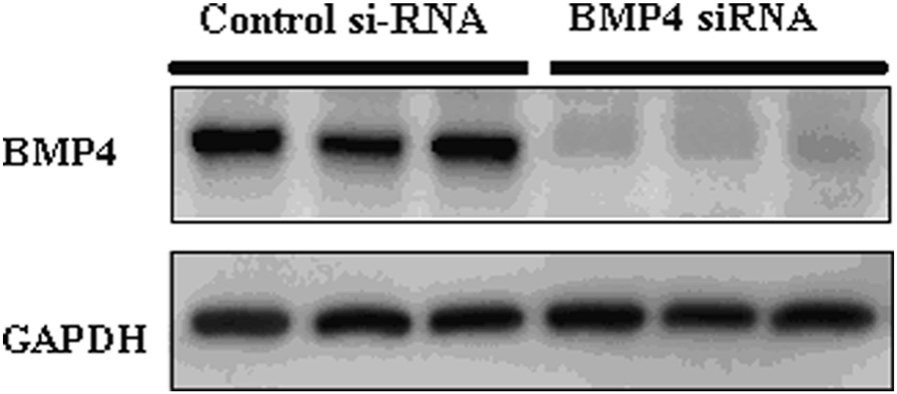
**The BMP4 expression in cardiomyocytes after si-RNA transfection.** BMP4 si-RNA transfection dramatically inhibited the BMP4 expression compared to the control si-RNA.

In the *in vitro* study, the BMP4 inhibition by si-RNA was confirmed by western blot assay (Figure 2). BMP4 si-RNA transfection dramatically inhibited the BMP4 expression compared to the control si-RNA.

**Figure 3:**
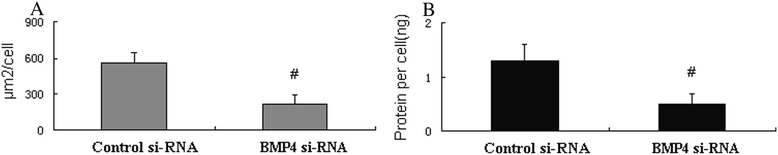
**BMP4 si-RNA transfection on the AngII induced cellular surface and cellular protein synthesis in cardiomyoctyes.** AngII dramatically increased the cellular surface in cardiomyoctye transfected with control siRNA. However, in cells transfected with BMP4 si-RNA, the cellular surface was markedly smaller than that in control si-RNA transfected cells **(A)** Similarly, the cellular protein synthesis dramatically decreased by BMP4 si-RNA transfection compared to control si-RNA transfection **(B)**.

AngII dramatically increased the cellular surface in cardiomyoctye transfected with control siRNA. However, in cells transfected with BMP4 si-RNA, the cellular surface was markedly smaller than that in control si-RNA transfected cells (560 ± 78 vs. 223 ± 78 um^2^/cell, P < 0.001, Figure 3A) Similarly, the cellular protein synthesis dramatically decreased by BMP4 si-RNA transfection compared to control si-RNA transfection (1.3 ± 0.2 vs. 0.5 ± 0.2 ng/cell, P < 0.001, Figure 3B).

## Discussion

In the present study, we explored the correlations of the BMP4 gene polymorphisms and the serum BMP4 levels with the development of LVH among Chinese EH patients. We found that the 6007C > T polymorphism of the BMP4 gene and the serum BMP4 level were significantly associated with the risk to develop LVH. Our in vitro study shows that the BMP4 inhibition in cardiomyocyte by si-RNA technique significantly decreased the Ang II induced cardiomyocyte size and protein content per cell, indicating the importance of BMP4 in the cardiomyocyte hypertrophy. Collectively, our data suggest that both the 6007C > T of the BMP4 gene and the serum BMP4 level may be used as potential marker for LVH incidence among the EH patients.

Bone morphogenetic proteins are osteoinductive growth factors that play a key role in cell differentiation, proliferation, migration, development, and apoptosis. BMP4 has been linked to the receptor-activator of nuclear factor-κB ligand (RANKL) mediated calcification in vessel smooth muscle cells [21]. Additional reports suggest roles for BMP4 in endothelial cell dysfunction, inflammation, and hypertension [15,22,28].

Several studies about BMP4 in cardiovascular system have implied that BMP4 might be involved in pathological cardiac hypertrophy, for example, BMP4 stimulates ROS production through NADPH oxidases in endothelium, exaggerates cardiac ischemia-reperfusion injury by promoting cardiomyocytes apoptosis [17]. BMP4 was involved in valvular interstitial cell activation in human myxomatous mitral valve prolapse [29]. BMP4 induces cardiomyocyte hypertrophy and apoptosis through increasing nadph oxidase 4 expression and reactive oxygen species-dependent pathways [19]. BMP4 is expressed in pathological cardiac hypertrophy models and BMP4-mediated cardiomyocyte hypertrophy [30]. In this study, we found that the BMP4 inhibition by si-RNA technique significantly blunt the AngII induced the hypertrophy of cardiomyocyte *in vivo*, supporting the role of BMP4 in the regulation of hypertrophic response of cardiomyocytes.

The single nucleotide polymorphism of 6007C > T (rs17563) of *BMP4* is the only identified polymorphism in the coding region [31]. The 6007 C > T polymorphism results in an amino acid change from valine to alanine at residue 152 (p.Val152Ala), thus affects the *BMP4* gene expression. The T-allele was predicted to change mRNA structure and the BMP4 mRNA levels were significantly higher in T-allele carriers compared with C-allele carriers, even the BMP4 protein plasma levels were higher among T-allele carries, but without reaching the statistical significance [32]. A recent study A significant association was observed between the 455C allele of BMP4 and increased left ventricular internal diameter systolic (p = 0.004) and between 1650 T allele of BMPR1B and lower left atrium diameter (p = 0.038). Presence of the 455C allele of *BMP4* and the 8474 T allele of ACVR1 gene was significantly associated with decreased left ventricular ejection fraction (LVEF) (p = 0.0004 and p = 0.046, respectively). The 455C allele of *BMP4* plays a role as significant predictors for decreased LVEF in newborns in newborns [33]. In this study, we found that the single nucleotide polymorphism of 6007C > T is significantly associated with the incidence of LVH in hypertensive patients, suggesting this genetic polymorphism can be used as molecular marker for LVH development. To the best of our knowledge, this is the first study reporting the role of *BMP4* genetic polymorphism with the LVH.

We studied several well established cytokine makers for prediction of LVH including cystatin C, PIIINP and hr-CRP. Serum cystatin C levels can be used to predict morbidity and mortality in patients with cardiovascular disease. A recent study shows that Serum cystatin C levels were higher in hypertensive patients with LVH than in those without LVH [34,35]. A community-based prospective cohort study reveals that serum PIIINP levels were higher in the presence of LVH than with no LVH in in hypertensive patients [36]. An independent risk factor for left ventricular hypertrophy in patients with lupus nephritis [37]. A positive association of left ventricular hypertrophy with high-sensitive CRP in hemodialysis patients has been reported [38]. In our study, we found that the 6007CC genotype carries had higher serum BMP4, cystatin C, PIIINP and hs-CRP levels. Correlation analyses reveal serum BMP4 level was significantly correlated to the serum cystatin C and PIIINP. We did not observed a positive correlation between hs-CRP and LVH in hypertensive patients in our study.

Several limitations should be addressed in this study. First, this is a single center based study and all the participants were ethnically Chinese in southeastern China area. Secondly, the sample size is relatively small. Secondly, we did not perform the functional role of *BMP4* 6007C > T mutation in cardiomyocytes due to the unavailability of *BMP4* mutation plasmid.
